# Saliva Metabolomics in Dry Mouth Patients with Head and Neck Cancer or Sjögren’s Syndrome

**DOI:** 10.3390/cells11030323

**Published:** 2022-01-19

**Authors:** Håvard Hynne, Elise Mørk Sandås, Katja Benedikte Prestø Elgstøen, Helge Rootwelt, Tor P. Utheim, Hilde Kanli Galtung, Janicke Liaaen Jensen

**Affiliations:** 1 Department of Oral Surgery and Oral Medicine, Faculty of Dentistry, University of Oslo, 0317 Oslo, Norway; j.c.l.jensen@odont.uio.no; 2 Department of Medical Biochemistry, Oslo University Hospital, 0424 Oslo, Norway; moerel@ous-hf.no (E.M.S.); kelgstoe@ous-hf.no (K.B.P.E.); hrootwel@ous-hf.no (H.R.); utheim2@gmail.com (T.P.U.); 3 Institute of Oral Biology, Faculty of Dentistry, University of Oslo, 0316 Oslo, Norway; h.k.galtung@odont.uio.no

**Keywords:** radiotherapy, head and neck cancer, Sjögren’s syndrome, saliva, metabolomics, pyrimidine signaling, purinergic receptors, amino acid metabolism

## Abstract

The etiology of dry mouth conditions is multi-faceted. Patients radiated after head and neck cancer (HNC) and those with primary Sjögren’s syndrome (pSS) share many of the same symptoms despite different causes. With the aim of better understanding the pathophysiology and biochemical processes behind dry mouth with different etiologies, we investigated the metabolic profile of 10 HNC patients, 9 pSS patients and 10 healthy controls using high-performance liquid chromatography-high resolution mass spectrometry (HPLC-MS) metabolomics. Principal component analysis (PCA) revealed different metabolic profiles when comparing all subjects included in the study. Both patient groups showed higher ratios of several pyrimidine nucleotides and nucleosides when compared to controls. This finding may indicate that purinergic signaling plays a role in dry mouth conditions. Moreover, significantly increased levels of DL-3-aminoisobutyric acid were found in HNC patients when compared to controls, and a similar tendency was observed in the pSS patients. Furthermore, a dysregulation in amino acid metabolism was observed in both patient groups. In conclusion, metabolomics analysis showed separate metabolic profiles for HNC and pSS patients as compared to controls that could be useful in diagnostics and for elucidating the different pathophysiologies. The demonstrated dysregulation of pyrimidine nucleotides and levels of metabolites derived from amino acids in the patient groups should be studied further.

## 1. Introduction

Dry mouth may lead to deteriorated oral health, including caries, Candida infection, distorted taste, and pronounced difficulties with speech and swallowing, severely reducing the person’s quality of life. Dry mouth affects >95% of head and neck cancer (HNC) patients treated with radiotherapy and patients with the autoimmune disease primary Sjögren’s syndrome (pSS) [1,2]. However, tissue damage after irradiation in HNC and autoimmune-induced salivary gland destruction in pSS represent different etiologies of dry mouth affliction [3]. When applying radiotherapy to HNC patients, doses above 20 gray (Gy) can cause damage to the salivary glands [4]. As can autoimmune-induced inflammation in pSS, where a gradual destruction of the salivary glands is observed.

The current management of dry mouth includes frequent sipping of water, saliva stimulants or saliva substitutes to increase the moisture in the mouth and lubricate the oral mucosa. Parasympathetic impulses provide the main stimulus for secretion of saliva by the secretory cells. Thus, muscarinic agonists have been used as saliva stimulants when some functional salivary gland tissue is present [5]. Additionally, purinergic receptors have recently been suggested as therapeutic targets to increase salivary secretion [1].

Unfortunately, existing management strategies to moisten a dry mouth offer temporary relief only. Furthermore, salivary substitutes lack the constituents that contribute to the protective effects of saliva [5]. In order to develop improved therapeutic solutions for salivary gland hypofunction, a better understanding of the pathophysiology and biochemical processes involved herein are crucial.

In recent years, many omics technologies have been applied to analyze salivary constituents, such as proteomics and transcriptomics [6,7,8]. Metabolomics is a rather new addition to the omics field, and involves the study of metabolites within biofluids, cells, and tissues. A metabolite is defined as a small molecule with a molecular weight typically less than 1500 Da [9]. These small molecules are the substrates, intermediates, and end products of biochemical reactions. The concentration of such molecules depends on the genetic properties of the organism and the environmental exposure, all of which influence the physiological or pathological state of the cell, tissue, or organism [10]. Metabolomics is a promising and powerful analytical tool. The improvements in high-performance liquid chromatography-mass spectrometry (HPLC-MS) in the last decade has allowed for the identification of thousands of metabolites in samples [11]. Thus, by using metabolomics, single molecules, ratios of metabolites, or patterns of metabolites, the biochemical pathways affected in diseases may be identified. In turn, these can be used as biomarkers for diagnosis, prognosis, and monitoring of disease progression and therapeutic effects. Furthermore, results from metabolomics can provide insight into the pathophysiology of a disease and could indicate new targets for therapeutic intervention. 

Until now, the application of metabolomics in dry mouth research has been limited to investigating potential biomarkers for pSS in saliva, urine, and blood [12,13,14,15]. A diversity of metabolites has been observed in these studies reflecting dysregulation in amino acid metabolism. However, there is still a paucity of data regarding whether the dysregulation is caused by the disease itself or is merely a consequence of hyposalivation [13]. 

In the present study, we aimed to establish a better understanding of the pathophysiology and biochemical processes behind dry mouth. By comparing two different patient groups suffering from dry mouth, we sought to identify the biochemical pathways that can be used to discriminate between patient groups and provide targets for further analyses of mechanisms.

## 2. Materials and Methods

### 2.1. Study Population and Design

This cross-sectional study is part of a larger research project performed as a collaboration between the Faculty of Dentistry, University of Oslo, and the Department of Medical Biochemistry, Oslo University Hospital. Samples were collected at the Dry Mouth Clinic at the Institute of Clinical Dentistry, Faculty of Dentistry, University of Oslo in the period from October 2015 to February 2019. The Norwegian Regional Committee for Medical and Health Research Ethics approved the study protocols (REK 2015/363 and 2018/1313) and the study was performed in compliance with the tenets of the Declaration of Helsinki. Written informed consent was obtained from all subjects prior to participation in the study. 

**Figure 1:**
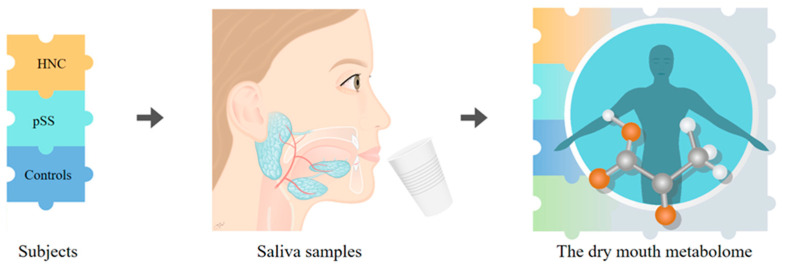
Graphical description of the study design. HNC—head and neck cancer patients; pSS—primary Sjögren’s syndrome patients. Figure produced by Sara Nøland.

The patients and controls included in the present study were selected from the larger project mentioned above, and the number of cases included was determined by the number of age- and gender-matched samples available. The following subjects were included: 10 HNC patients who had undergone radiotherapy, nine patients diagnosed with pSS, fulfilling the American–European Consensus Group classification criteria [16], and 10 healthy controls without symptoms of dryness. Due to the low prevalence of pSS in men [17], only females were included. To the best of our knowledge, the subjects had no other diseases known to cause sicca symptoms and did not use medications influencing saliva production. Figure 1 presents a graphical description of the study design.

All HNC patients had been treated with radiotherapy at the Department of Oncology, Oslo University Hospital, Norway, and reported problems related to dry mouth. All patients received postoperative radiotherapy (total dose of 50–70 Gy) delivered as 2 Gy per fraction and administered 5–6 times per week. The patient group is fully described in Westgaard et al. [18].

Specialists in rheumatology referred the pSS patients to the Department of Oral Surgery and Oral Medicine, Faculty of Dentistry, University of Oslo. Information collected during routine laboratory assessments was provided, including anti-Ro/SSA and anti-La/SSB, as well as values for saliva and tear secretion. Some residual secretory ability was required for inclusion in the study to enable sample collection. All patients fulfilled the 2002 criteria for pSS [16]. The patient group is fully described in Tashbayev et al. [19].

Demographic characteristics of the study subjects are summarized in Table 1. All study subjects were female, and the groups were matched according to age, ethnicity, smoking status, educational level, and occupational status.

### 2.2. Patient-Reported Outcomes and Examination of Dry Mouth

All subjects underwent subjective and objective dry mouth evaluation. The examinations were conducted at The Dry Mouth Clinic at the Faculty of Dentistry, University of Oslo. The subjective measure for dry mouth was the Summated Xerostomia Inventory-Dutch Version, and the objective measure was the Clinical Oral Dryness Score index [18,20]. All subjects were instructed to refrain from eating, drinking, and smoking 1 h prior to their appointment. The examinations were performed by a team of experienced dentists and dental specialists.

### 2.3. Saliva Sample Collection and Sample Preparation

Unstimulated whole saliva (UWS) and chewing-stimulated whole saliva (SWS) were collected according to a standardized predefined protocol previously described [6]. Strict routines were employed to ensure standardization of the method for saliva collection. In brief, all saliva samples were chilled on ice during collection, and the saliva was collected in plastic cups weighed to the nearest decigram. For UWS, the subjects first swallowed all saliva in the mouth. Thereafter, they avoided swallowing and were instructed to for 15 min regularly spit all saliva produced into a plastic cup. SWS was collected while the patients chewed on a paraffin pellet (Ivoclar Viavadent, Shaen, Lichenstein). After an initial chewing period of approximately 30 s, the subjects were asked to swallow all saliva and then continue chewing for five minutes. During the five minutes, the patients were asked to not talk and were instructed to spit regularly into the plastic cup. Following the collection of UWS and SWS, the salivary secretion rates were calculated before freezing at −80 °C.

Due to the low amount of UWS collected, and the high viscosity of the samples, SWS was chosen for the metabolomics analysis. The samples were thawed at room temperature and vortexed. A 200 µL sample was transferred to a 0.22 µm cellulose acetate spin filter (Agilent (Santa Clara, CA, USA)) and centrifuged using Fresco 21 Microcentrifuge (Thermo Scientific (Waltham, MA, USA)) for 10 min at 14,000× *g*, (21,100 RCF) at 4 °C. The filtrate was transferred to an HPLC vial prior to metabolomics analysis. To correct for analytical drift and ensure high quality of the metabolomics data collected, pooled group samples were made by mixing equal volume of all samples in a group. Equal volume of the pooled group samples was then mixed to make a pooled quality control (PQC) sample. The PQC was analyzed repeatedly throughout the sample batch and used for signal corrections. A blank sample (LC-MS grade water) was prepared in the same manner as the saliva samples.

### 2.4. Metabolomics Analyses

Metabolomics analysis was performed using a previously described, validated in-house method for global metabolomics [21]. The sample preparation method was different due to other sample material used. pSS, HNC, control, PQC, and blank samples were analyzed using fullMS mode in random order. The pooled group samples were analyzed using ddMS2 mode. The PQC sample was analyzed between every fifth sample. All samples were analyzed using both positive and negative electrospray ionization mode in separate injections.

## 3. Database and Statistics

### 3.1. Statistical Software 

Compound Discoverer 3.1 (from Thermo Scientific) was used for data processing and statistical analyses using the workflow template: ‘Untargeted Metabolomics with Statistics Detect Unknowns with ID using Online Databases and mLogic’. The statistical analyses on the clinical parameters were performed with the commercial software SPSS for Windows, version 26 (IBM, Chicago, IL, USA). One-way ANOVA with Bonferroni Post Hoc when applicable was used in the intergroup comparison of parameters. A *p*-value of <0.05 was chosen as significant. There were no missing data in the dataset.

### 3.2. Metabolite Identification and Interpretation 

Compound Discoverer utilized the following databases for metabolite identification: the ChemSpider (http://www.chemspider.com//) (accessed on 22 September 2021) database was used to search FullMS scans by using the molecular weight or predicted formulas when available. The mzCloud (https://www.mzcloud.org/) (accessed on 22 September 2021) database was used to search MSMS scans by using the fragmentation pattern, molecular weight, or predicted formulas when available.

For the post-analytical interpretation, the Human Metabolome Database (https://hmdb.ca/) (accessed on 25 October 2021) was used. An explanation of the level of identification is provided in Table 2.

## 4. Results

### 4.1. Clinical Features

Clinical examinations at the Dry Mouth Clinic, Faculty of Dentistry, revealed more pronounced symptoms and clinical findings of dry mouth in HNC and pSS patients as compared to controls. The study subjects’ salivary secretion rates are summarized in Table 3. Unsurprisingly, there were significant intergroup differences in the salivary secretion. However, saliva volumes were significantly different only between pSS patients and controls.

### 4.2. HPLC-MS Metabolomics Analysis

The global metabolomics analysis revealed 2853 features using positive (ESI+) and 851 features using negative (ESI−) electrospray ionization modes. Some features identified as components of plastic were common in 6 of 9 pSS samples and were excluded in further analyses. 

### 4.3. Principal Component Analysis

**Figure 2:**
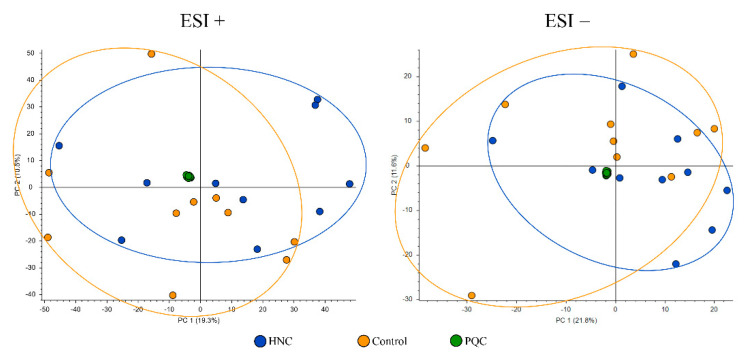
Principal component analysis plot of salivary metabolites in head and neck cancer patients (HNC) and controls. PQC—pooled quality control; ESI+—positive electrospray ionization; ESI−—negative electrospray ionization; and PC—principal component. Ellipses show sample distributions.

**Figure 3:**
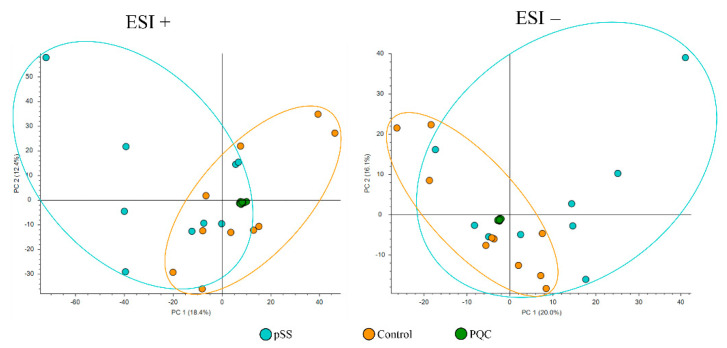
Principal component analysis plot of salivary metabolites in primary Sjögren’s syndrome patients (pSS) and controls. PQC—pooled quality control; ESI+—positive electrospray ionization; ESI−—negative electrospray ionization; and PC—principal component. Ellipses show distribution of the samples.

**Figure 4:**
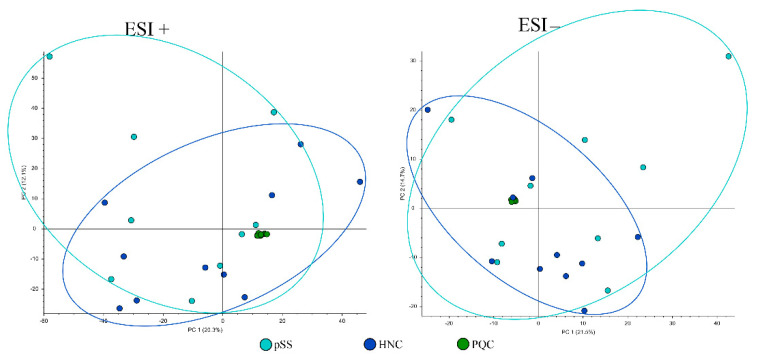
Principal component analysis plot of salivary metabolites in head and neck cancer patients (HNC) and primary Sjögren’s syndrome patients (pSS). PQC—pooled quality control; ESI+—positive electrospray ionization; ESI−—negative electrospray ionization; and PC—principal component. Ellipses show sample distributions.

An overview of the analysis quality was obtained by including the PQC in the principal component analyses (PCA). As shown in the PCA plots (Figure 2, Figure 3 and Figure 4), different metabolic profiles were found when comparing all subjects included in the study and all PQC samples were very well grouped, demonstrating the high quality and low imprecision of the analyses. Furthermore, we only included components where the PQC variated less than 30%.

PCAs of HNC patients compared to controls in both positive and negative electrospray ionization are provided in Figure 2. PCA scores ESI+: PC 1 = 19.3%, PC 2 = 10.5% and PCA scores ESI−: PC 1 = 21.8%, PC 2 = 11.6%.

PCA plots of pSS patients compared to controls in both positive and negative electrospray ionization are shown in Figure 2. PCA scores ESI+: PC 1 = 18.4%, PC 2 = 12.4% and PCA scores ESI−: PC 1 = 20.0%, PC 2 = 16.1%.

PCA plots of HNC patients compared to pSS patients in both positive and negative electrospray ionization are shown in Figure 2. PCA scores ESI+: PC 1 = 20.3%, PC 2 = 12.1% and PCA scores ESI−: PC 1 = 21.5%, PC 2 = 14.7%.

### 4.4. Metabolite Identification and Ratios

Before post-analytical interpretation and further identification based on retention time and reference standard, only molecular features with *p*-values less than 0.05 and ratios higher than two or below 0.5 were selected for further identification and interpretation. 

After choosing relevant features, a total of 66 and 17 metabolites were identified in positive and negative electrospray ionization modes, respectively (Table 4). A total of 13 metabolites had *p*-values less than 0.05 and a ratio higher than two or below 0.5 in both HNC and pSS when compared to controls.

**Figure 5:**
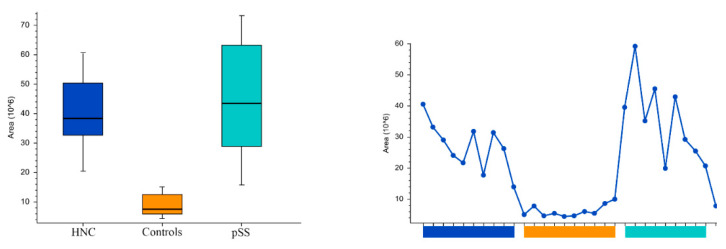
The peak areas of cytosine for the groups are shown in box plot to the left and, and for the individual samples to the right. HNC—head and neck cancer patients; pSS—primary Sjögren’s syndrome patients.

**Figure 6:**
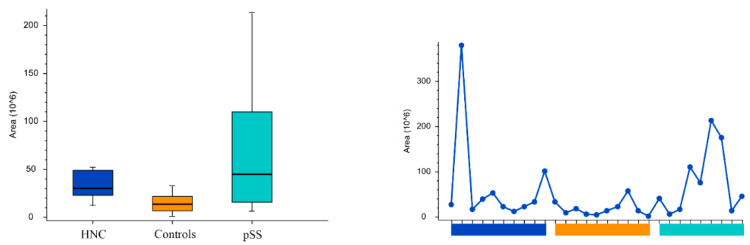
The peak areas of uridine for the groups are shown in box plot to the left and for the individual samples to the right. HNC—head and neck cancer patients; pSS—primary Sjögren’s syndrome patients.

**Figure 7:**
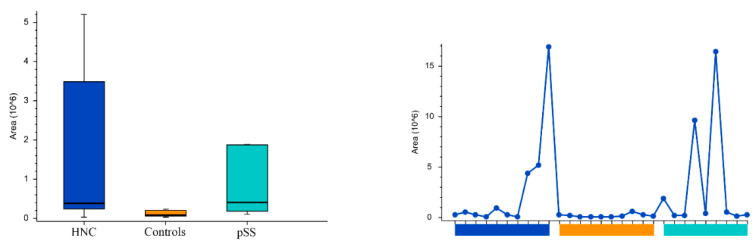
The peak areas of cytidine5′-monophosphate for the groups are shown in box plot to the left and for the individual samples to the right. HNC—head and neck cancer patients; pSS—primary Sjögren’s syndrome patients.

**Figure 8:**
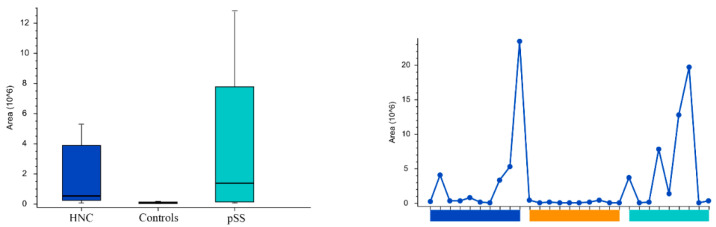
The peak areas of uridine monophosphate for the groups are shown in box plot to the left and for the individual samples to the right. HNC—head and neck cancer patients; pSS—primary Sjögren’s syndrome patients.

**Figure 9:**
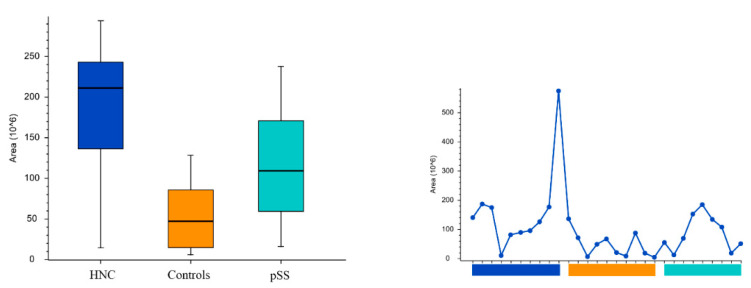
The peak areas of DL-3-Aminoisobutyric acid for the groups are shown in box plot to the left and for the individual samples to the right. HNC—head and neck cancer patients; pSS—primary Sjögren’s syndrome patients.

Peak areas of the groups and the individual samples for the pyrimidine nucleotides and nucleosides cytosine, uridine, cytidine5′-monophosphate, and uridine monophosphate are shown in Figure 5, Figure 6, Figure 7 and Figure 8, respectively. Peak areas of the groups and the individual samples for DL-3-Aminoisobutyric acid are shown in Figure 9.

**Figure 10:**
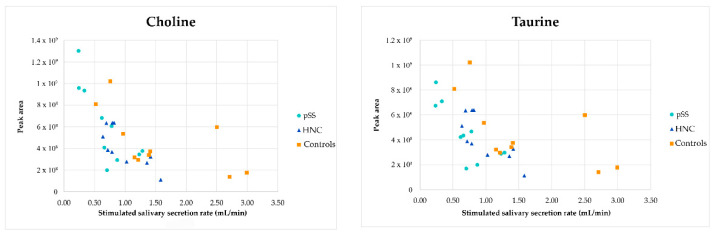
Relationship between peak area and stimulated salivary secretion rate for choline and taurine. HNC—head and neck cancer patients; pSS—primary Sjögren’s syndrome patients.

The relationship between the two metabolites choline and taurine and SWS is shown in Figure 10. A negative correlation between the amount of metabolites present and SWS can be visualized for all subjects, not only the patient groups.

## 5. Discussion and Conclusions

The present study marks the first time that metabolic profiles of saliva have been simultaneously explored in two patient groups suffering from dry mouth compared to a healthy control group without dryness symptoms. Here, we show different metabolic profiles of the two patient groups suffering from dry mouth and distinct differences between the patient groups and controls. These findings indicate that the two patient groups have unique metabolic profiles. 

Several nucleotides and nucleosides were found in higher ratios in the patient groups when compared to controls. Both uridine/uridine monophosphate and cytosine/cytidine 5′-monophosphate belong to the group of pyrimidine nucleotides. Nucleotides are nucleosides with phosphate groups attached and are the building blocks of nucleic acids. Besides their function as nucleic acids, pyrimidine nucleotides play an important part in cellular metabolism. Additionally, both uridine monophosphate and cytidine 5′-monophosphate may function in purinergic receptor signaling and as intracellular second messengers [25]. Pyrimidine nucleotides are initially metabolized to nucleosides by pyrimidine nucleotidases, that may eventually be broken down to aminoisobutyric acid. Interestingly, significantly increased levels of DL-3-aminoisobutyric acid were found in HNC patients when compared to the controls. A similar tendency of DL-3-aminoisobutyric acid could be seen in the pSS patient samples, but no statistical significant differences were found.

The metabolomic analysis performed in the present study revealed higher ratios of uridine, uridine monophosphate, and cytidine 5′-monophosphate in the patient groups when compared to the controls. Cytosine was found in higher ratios in pSS patients compared to controls, potentially indicating that decreased purinergic signaling may play a role in the pathophysiology of salivary hypofunction. The P2 purinergic receptors are important for many physiological processes in numerous tissues, including the salivary glands. Interestingly, a P2Y receptor agonist is currently in use for the treatment of dry eye disease, and purinergic receptors have recently been suggested as therapeutic targets to increase salivary secretion [1,26]. Topical administration of the P2Y receptor agonist uracil-cytosine dinucleotide promotes fluid and mucin secretion in the eyes, and a meta-analysis concluded that it may be effective in the treatment of dry eye disease [26]. Knowing that both HNC and pSS patients may suffer from dry eyes and dry mouth [18,20], the potential role of a P2Y receptor agonist should be investigated further in both conditions. The P2Y receptors are reported to be upregulated upon damage to the salivary glands and in salivary glands of Sjögren’s syndrome mouse models [27]. These findings suggest that pyrimidine pathways play a role in conditions where salivary glands are damaged and should therefore be evaluated as a future therapeutic target. Additionally, further investigation of the role of such receptors, or their upregulation, in patients with compromised salivary glands could be a goal for future research.

Interestingly, the levels of gamma-glutamyl-leucine were found in a higher ratio in pSS patients compared to HNC patients. Gamma-glutamyl-leucine is among the key constituents of the glutamyl cycle and the synthesis of glutathione [28]. Glutathione is an antioxidant and has been linked to the development and progression of several diseases, such as cancer, rheumatoid arthritis, insulin-dependent diabetes mellitus, and multiple sclerosis [29]. The pyrimidine signaling and glutathione networks are closely related and regulate inflammatory processes [30]. These findings further indicate a role of pyrimidine signaling in conditions causing damage to the salivary glands.

An additional interesting finding when comparing the two patient groups to controls was the dysregulated levels of several metabolites derived from amino acids in the pSS patients. Many of the metabolites identified were dipeptides and Ochoa et al. reported similar results in a metabolic analysis of urine from pSS patients [14]. A disturbance of amino acid metabolism has previously been linked to pSS and changes in salivary flow [14,15]. Moreover, Mondanelli et al. suggested amino acid metabolism as a potential drug target in autoimmune diseases [31]. Because both patient groups were suffering from damage to the salivary gland, this dysregulation may indicate a relationship between the disturbed amino acid metabolism and the immune-mediated damage seen in pSS. Mikkonen et al. reported a significantly higher concentration of taurine and choline in pSS patients compared to healthy controls and a negative correlation of these metabolites with the salivary flow rate [15]. In the present study, a similar negative correlation was found for all subjects investigated, not only the patient groups. Moreover, there was no significant difference in taurine and choline concentrations when comparing the patient groups with controls in the present work. One could argue that differences in salivary secretion rates between pSS patients and controls may partly explain variance in the amount of metabolites present. Theoretically, the metabolite concentration in the patient groups could be due to reduced salivary secretion, increased metabolite production, or a combination of these. However, no statistically significant differences in salivary secretion rates were found between HNC and pSS patients or between HNC patients and controls, underlining that most of the results were unrelated to salivary secretory rate. Furthermore, we acknowledge other possible sources than salivary glands for metabolites in saliva, such as exogenous compounds and sloughing from both eukaryotic and prokaryotic cells.

In addition to the metabolites identified with their name and function, the majority of the metabolites that were significantly altered in amounts were not identified with their unique name and function. This is a well-known limitation when utilizing HPLC-MS in global metabolomics, and only ~10% of known metabolites have experimental spectral data in databases [32]. Consequently, several features could not be identified and were named by their molecular mass in the results. Further development of spectral databases will improve this situation in the future. 

All subjects in the study were examined by the same personnel following the same protocol, and all samples were, to the best of our knowledge, handled, stored, and treated identically. Furthermore, the subjects were matched according to age, ethnicity, smoking status, educational level, and occupational status. This approach reduces unwanted noise and variance in the data and is of utmost importance when utilizing sensitive analytical methods such as HPLC-MS metabolomics. 

In conclusion, we showed separate metabolic profiles for HNC and pSS patients as compared to controls that could be useful for elucidating the differences in pathophysiology in groups suffering from dry mouth. The demonstrated dysregulation of pyrimidine nucleotides and levels of metabolites derived from amino acids in the patient groups remain to be investigated further. Furthermore, because the available metabolite databases continually become more comprehensive, many of the metabolites in this study will be uniquely identified and may provide new and better biomarkers and point to new potential therapeutic targets.

## Figures and Tables

**Table 1:** Summary of subject characteristics. Values are presented as the mean ± SD or percentage. HNC—head and neck cancer patient; pSS—primary Sjögren’s syndrome. Intergroup comparison was performed using ANOVA.

Characteristics	HNC (n = 10)	pSS (n = 9)	Controls (n = 10)	*p*-Value
	Mean ± SD	Mean ± SD	Mean ± SD	
Age (years)	59.1 ± 8.5	53.2 ± 13.9	53.7 ± 2.3	0.3
	%	%	%	
Ethnicity				0.4
Scandinavian	100%	100%	90%	
Other			10%	
Smoking status				0.1
Yes	30%	11%	0%	
No	70%	89%	100%	
Education level				0.7
Basic	0%	0%	0%	
Secondary	10%	20%	10%	
Higher	90%	80%	90%	
Occupation				0.2
Working	40%	60%	100%	
Sick leave	50%	20%	0%	
Student	0%	0%	0%	
Retired	10%	20%	0%	

**Table 2:** Explanation of level of identification.

Level of ID	Identification
Level 1	Validated identification using in-house library (MS/MS spectrum and retention time match).
Level 2	Putative identification using online databases (MS/MS spectrum match).
Level 3	Putative identification supported by additional information.
Level 4	Tentative identification using online databases (chemical formula).
Level 5	Unique feature. Molecular mass ± 5 ppm.

**Table 3:** Mean values and ± SD. HNC—head and neck cancer patients; pSS—primary Sjögren’s syndrome; UWS—unstimulated whole saliva (mL/min); SWS—stimulated whole saliva (mL/min). Intergroup comparisons were carried out by performing ANOVA with Bonferroni Post Hoc test between the groups of subjects. ^a^ Significant difference between pSS and controls, *p* < 0.05.

Clinical Parameter	HNC (n = 10)	pSS (n = 9)	Controls (n = 10)	*p*-Value
	Mean ± SD	Mean ± SD	Mean ± SD	
UWS (mL/min) ^1^	0.13 ± 0.1	0.09 ± 0.07 ^a^	0.27 ± 0.23 ^a^	0.03
SWS (mL/min) ^2^	1.0 ± 0.3	0.7 ± 0.4 ^a^	1.6 ± 0.9 ^a^	0.01

^1^ Normal unstimulated salivary secretion rate: 0.3–0.4 mL/min [22,23]. ^2^ Normal stimulated salivary secretion rate: 1.5–2 mL/min [24].

**Table 4:** HNC—head and neck cancer patients; pSS—primary Sjögren’s syndrome. E—electro spray ionization. ↑↑: ratio > 10; ↑: ratio 2–9.9; ↓: ratio 0.1–0.5; ↓↓: ratio < 0.1. * Features that could not be identified are named by their molecular mass ± 5 ppm. ** Metabolites with *p*-values less than 0.05 and a ratio higher than two or below 0.5 in both HNC and pSS when compared to controls. +: positive, −: negative.

Name	Level	Ratio: HNC/Controls	Ratio: pSS/Controls	Ratio: HNC/pSS	ESI
Pyrogallol **	4	↑	↑	↓	+
O-Phosphorylethanolamine	1	↑↑	↑		−
319.99404 *^,^**	5	↑↑	↑		−
163.00087 *^,^**	5	↑	↑↑		+
Uridine monophosphate **	1	↑	↑↑		−
134.99907 *^,^**	5	↑	↑↑		−
Streptidine **	4	↑	↑		+
Vanillin **	2	↑	↑		+
178.97480 *^,^**	5	↑	↑		+
Vanillin **	2	↑	↑		−
Creatine **	1	↑	↑		−
Cytidine 5′-monophosphate **	1	↑	↑		−
Uridine **	1	↑	↑		−
Υ-L-Glutamyl-L-glutamic acid **	2	↓	↓		+
N-Tridecanoylglycine	4	↑↑		↑↑	−
(E)-2-[(2S)-2-Amino-2-carboxyethoxy]-2-hydroxyethenediazonium	4	↑↑			+
N-Acetylvaline	4	↓↓			+
Xylitol	2	↑		↑	−
DL-Stachydrine	2	↓		↓	+
Xylitol	1	↑			+
DL-3-Aminoisobutyric acid	1	↑			+
282.03789 *	5	↑			+
194.07065 *	5	↑			+
Butylparaben	4	↑			−
Diethylene glycol	4	↓			+
2,2′-[1,2-Propanediylbis(oxy)]diethanol	4	↓			+
4-Morpholinylacetic acid	4	↓			+
499.26496 *	5	↓			+
474.54143 *	5	↓			+
Hydroxychloroquine	2		↑↑	↓↓	−
Hydroxychloroquine	2		↑↑	↓↓	+
Cytosine	1		↑↑		+
214.61102 *	5		↓↓		+
Monodesethylchloroquine	2		↑	↓↓	+
2-Aminoadipic acid	1		↑	↑	−
N-(1-{[Methyl(2-methyl-2-propanyl)carbamoyl]amino}ethyl)-alpha-asparagine	4		↑	↓	+
asn-val	4		↑	↓	+
Meprobamate	4		↑	↓	+
Threonylphenylalanine	4		↑	↓	+
225.07485 *	5		↑	↓	+
396.23525 *	5		↑	↓	+
Pantothenic acid	4		↑		−
Paraldehyde	4		↑		−
Pyr-Val-OH	4		↑		−
345.09776	5		↑		−
1-Methylnicotinamide	1		↑		+
Tyrosylalanine	2		↑		+
gamma-L-glutamyl-L-tyrosine	4		↑		+
Gly-Leu	4		↑		+
Leucylasparagine	4		↑		+
Phenylalanylproline	4		↓		+
L-Alanyl-L-glutamine	2		↓		+
127.02446 *	5		↑		+
324.03541 *	5		↑		+
459.26897 *	5		↑		+
Asp-Val	2			↓	+
Gly-Phe	2			↓	+
L-gamma-Glutamyl-L-leucine	2			↓	+
Phenylalanylalanine	2			↓	+
Threonylleucine	2			↓	+

## Data Availability

The datasets generated and/or analyzed during the current study are not publicly available due to ethical restrictions enforced by the research and medical institutions under license for the current study. Data are, however, available from the authors upon reasonable request and with permission of the Regional Medical Ethical Committee of South-East Norway, the University of Oslo and Oslo University Hospital.
